# Fabrication and Characterization of Phyllanthus Emblica Extract-Polyvinyl Alcohol/Carboxymethyl Cellulose Sodium Antioxidant Hydrogel and Its Application in Wound Healing

**DOI:** 10.3390/pharmaceutics16121531

**Published:** 2024-11-29

**Authors:** Shanqin Huang, Shanglun Li, Guoyan Li, Chenyu Wang, Xiaohan Guo, Jing Zhang, Jing Liu, Ying Xu, Yanchun Wang

**Affiliations:** 1 College of Pharmacy, Jiangsu University, Zhenjiang 212013, China; 18852891267@163.com (S.H.);; 2 Henan Provincial People’s Hospital, Zhengzhou 450003, China; 3 School of Medical, Henan University of Traditional Chinese Medicine, Zhengzhou 450046, China

**Keywords:** Phyllanthus emblica, polyphenol, gallic acid, PVA/CMC-Na hydrogel, sustained release, antioxidant, anti-inflammatory, wound healing

## Abstract

**Background**: Phyllanthus emblica is a medicinal and edible plant from the Euphorbiaceae family, notable for its rich content of polyphenols and flavonoids, which provide significant antioxidant properties. To exploit the full antioxidant potential of Phyllanthus emblica, this study developed a hydrogel system incorporating polyvinyl alcohol (PVA) and carboxymethyl cellulose sodium (CMC-Na), integrated with Phyllanthus emblica extract, for the purpose of wound healing. **Methods**: The extraction process of active ingredients of Phyllanthus emblica was optimized and assessed the antioxidant composition and activity of the extract. A series of hydrogel performance evaluations were performed on the Phyllanthus emblica extract-loaded PVA/CMC-Na hydrogel (AEPE composite hydrogel). Additionally, the wound healing efficacy was evaluated through cell culture experiments and wound healing assays using BALB/C mice. **Results**: The findings indicated that the extraction of Phyllanthus emblica with 95% ethanol yielded an extract rich in polyphenols, primarily gallic acid and ellagic acid, demonstrating high free radical scavenging capacity and robust antioxidant activity. The hydrogel matrix containing 12% PVA and 1% CMC-Na exhibited excellent physicochemical properties. The optimized AEPE composite hydrogel enabled sustained drug release over a 24 h period, exhibited low cytotoxicity and promoted cell migration. In a mouse dorsal wound healing model, the AEPE composite hydrogel showed pronounced anti-inflammatory and antioxidation effects, enhanced collagen deposition, and ultimately accelerated wound healing. **Conclusions**: The AEPE composite hydrogel demonstrated strong antioxidant characteristics and significant wound healing potential. Thus, this study could broaden the application prospects of Phyllanthus emblica in wound healing.

## 1. Introduction

Phyllanthus emblica, commonly known as Indian gooseberry or amla, is a medicinal and edible plant belonging to the Euphorbiaceae family. The dried mature fruit of Phyllanthus emblica is extensively cultivated and widely utilized as both a food product and a therapeutic agent across several Asian countries, including India, China, and Malaysia. Historical records indicate that its medicinal use dates back to early traditional systems, such as references to “the south of grass”, underscoring its considerable therapeutic value [1]. Extensive research has elucidated that Phyllanthus emblica contains a diverse array of bioactive compounds, primarily phenolic compounds such as gallic acid, chlorogenic acid, and ellagic acid [2], as well as flavonoids, sesquiterpenoids, volatile oils, proteins, and essential minerals, including potassium, calcium, magnesium along with a wide range of vitamins [3]. The polyphenols and flavonoids inherent in Phyllanthus emblica exhibit significant antioxidant potential and hyaluronidase inhibitory activities [4]. As a result, Phyllanthus emblica demonstrates a wide spectrum of pharmacological activities, including antioxidative, anti-inflammatory, antibacterial, antitumor, liver-protective, anti-metabolic disorders, and cardioprotective activities [5,6,7]. Consequently, Phyllanthus emblica is frequently formulated into various preparations used in both pharmaceutical and cosmetic industries [8].

The antioxidant activity of Phyllanthus emblica is attributed to its ability to scavenge various free radicals or reactive oxygen species, thereby reducing oxidative stress damage and maintaining redox state stability [9,10]. Phyllanthus emblica has been reported to possess significant antioxidant capacity in iron reduction and lipid peroxidation inhibition, largely due to the conversion of free carboxyl groups in polyphenols to more potent oxidizing ellagic acid esters [4,11]. The polyphenol content in extracts obtained by different extraction methods varies significantly, and there remains limited research on the development and application of these antioxidant extracts. Wound sites are often characterized by oxidative stress imbalance, with elevated peroxide levels impairing the healing process [12]. Therefore, the antioxidant components in Phyllanthus emblica extract may provide considerable benefits in promoting wound healing, although research in this specific area remains sparse.

Traditional wound dressings protect wounds and promote healing but face limitations such as strong adhesiveness, inadequate moisture retention, and poor permeability [13,14]. These drawbacks significantly restrict their therapeutic application, prompting the need for novel wound dressings [15]. Among these, hydrogel dressings with their three-dimensional porous network structure exhibit excellent biocompatibility, biodegradability, and hydration capabilities, and have been widely utilized in trauma treatment [16,17]. Polyvinyl alcohol (PVA) is a biodegradable, non-toxic, hydrophilic polymer material. However, PVA hydrogel alone suffers from limitations such as insufficient elasticity, membrane rigidity, and incomplete hydrophilicity [18]. Carboxymethyl cellulose sodium (CMC-Na), a hydrogel material with robust moisture absorption and expansion capabilities, enhances the ductility and swelling performance of composite hydrogel [19]. Blending synthetic polymers with natural polymers results in hydrogels with superior mechanical properties compared to single-component systems [13].

In this study, we aimed to explore the antioxidant properties of Phyllanthus emblica and develop a novel PVA/CMC-Na hydrogel that harnesses its antioxidant properties for long-term therapy to accelerate wound healing. Active ingredients with antioxidant potential in Phyllanthus emblica were extracted using various methods to optimize the extraction process, and the main antioxidant components in the extracts were analyzed and identified, along with evaluations of its free radical scavenging and antioxidant activities. Additionally, the performance metrics of hydrogels with various ratios were examined to identify the optimal PVA/CMC-Na hydrogel loaded with Phyllanthus emblica extract, followed by an investigation into its antioxidant repair and wound healing capabilities.

## 2. Materials and Methods

### 2.1. Materials

Folin–Ciocalteu (Folinol), ABTS, and DPPH were purchased from Shanghai Macklin Biochemical Technology Co., Ltd. (Shanghai, China). Aluminum nitrate and CMC-Na were bought from the National Medicine Group Chemical Reagent Co., Ltd. (Shanghai, China). Gallic acid and rutin leaves were bought from Shanghai Biological Technology Co., Ltd. (Shanghai, China). PVA 1799 was bought from Yatai United Chemical Co., Ltd. (Wuxi, China). Other reagents and chemicals used were of analytical grade. Mice embryonic fibroblasts, 3T6—Swiss albino, were purchased from the cell bank of Chinese Academy of Sciences (Shanghai, China). BALB/C mice (male, 3–4 weeks old, weighing between 18 and 22 g) were purchased from the Laboratory Animal Center of Jiangsu University (Zhenjiang, China) and the protocol and procedures employed were ethically reviewed and approved.

### 2.2. Extracts of Phyllanthus Emblica with Different Solvents

After grinding the dried fruit into powder, pure water, 75% ethanol, and 95% ethanol were used as solvents, and the mixture was heated and extracted by reflux at a ratio of 1:10 (*w*/*v*). The combined extracts were concentrated under vacuum at 50 °C and freeze-dried (ethanol extracts were rotary evaporated to remove the ethanol) to obtain the water extracts (WEPE) and ethanol extracts (AEPE) of Phyllanthus emblica.

### 2.3. Determination of Total Polyphenol Content

The total polyphenol content of WEPE and AEPE was determined as described by Aksornsri et al. [20]. The lyophilized extract powder was weighed and diluted with the corresponding extraction solvent to obtain the sample solution (0.1 mg/mL). Folinol reagent and purified water were then added, followed by 7.5% Na_2_CO_3_ solution. The mixture was left to react, and the absorbance was measured at 765 nm using a UV/VIS spectrophotometer (UV/VIS-8000, Shanghai Metash Instruments Co., Ltd., Shanghai, China). The total polyphenol content was then calculated using the established standard curve.

### 2.4. Determination of Antioxidant Active Components in AEPE

High performance liquid chromatography (SPD, SHIMADZU CURPORATION, Kyoto, Japan) was employed to determine the active components in Phyllanthus emblica, following the method described by Gan et al. [21]. The concentrations of the three components were determined using a C18 column (4.6 mm × 250 mm, 5 μm) with a UV detection wavelength of 254 nm. The mobile phase consisted of solution A (methanol) and solution B (0.2% (*v*/*v*) phosphoric acid aqueous solution), and gradient elution was used. The gradient profile was as follows: 0–5 min, 5–10% (B); 5–15 min, 10–25% (B); 15–30 min, 25–30% (B); and 30–55 min, 30–95% (B). The flow rate was 0.6 mL/min, the column temperature was maintained at 30 °C, and the injection volume was 20 μL. Phenolic compounds were identified by comparing retention times with standards and by spiking samples with known standards.

### 2.5. DPPH Radical Scavenging Activity

The DDPH free radical scavenging assay was conducted on the AEPE in accordance with the method described by Guo et al. [22]. All samples were analyzed with a UV/VIS spectrophotometer at a wavelength of 517 nm. The sample (0.5 mg/mL) was mixed with DPPH–ethanol solution (0.1 mg/mL) and incubated for 1 h in the dark, after which absorbance was measured (A_1_). Ethanol was used in place of DPPH–ethanol solution to determine the control absorbance value (A_2_), and ethanol was used to instead of the sample to measure the blank absorbance value (A_0_). The DPPH radical scavenging rate is calculated as follows:

(1) $$Scavenging\;rate\left(\%\right)=\left(\frac{1-\left(A_{1}-A_{2}\right)}{A_{0}}\right)\times 100\%$$

### 2.6. ABTS^+^ Radical Scavenging Activity

The ABTS^+^ radical scavenging assay was conducted on the AEPE following the method described by Li et al. [11]. In brief, ABTS^+^ working solution was prepared by mixing equal volume of ABTS^+^ solution (7 mmol/L) and potassium persulfate solution (2.6 mmol/L). And 25 μL samples (0.5 mg/mL) were mixed with 3.0 mL ABTS^+^ working solution. All samples were detected by UV/VIS at a wavelength of 734 nm. After 6 min of incubation at room temperature in the dark, the absorbance was measured (A_1_). For the control, ethanol was used instead of the ABTS^+^ working solution to measure the absorbance (A_2_), and ethanol was used instead of the sample to measure the blank absorbance (A_0_). The ABTS^+^ radical scavenging rate is calculated as shown in Formula (1).

### 2.7. Hydroxyl Radical Scavenging Activity

The hydroxyl radical scavenging assay was conducted on the AEPE following the method described by Panchal et al. [23]. The sample solution (0.5 mg/mL) was mixed with salicylic acid–ethanol solution (9 mmol/L) and FeSO_4_ solution (9 mmol/L). Subsequently, H_2_O_2_ solution (8.8 mmol/L) was added to initiate the color reaction. The mixture was thoroughly mixed and incubated in a 37 °C water bath. All samples were detected by UV/VIS at a wavelength of 510 nm. For the blank, ethanol was used to measure the absorbance of the mixture (A_1_). Ethanol was also used in place of the H_2_O_2_ solution to measure the control absorbance (A_2_), and ethanol was used instead of the sample to measure the blank absorbance (A_0_). The hydroxyl radical scavenging rate is calculated as shown in Formula (1).

### 2.8. Preparation AEPE Loading PVA/CMC-Na Hydrogel

The PVA solution was prepared by weighing the indicated amount of PVA and AEPE, followed by mixing with 90 mL of 40% glycerol aqueous solution while heating and stirring at 130 °C. The CMC-Na solution was obtained by stirring a certain amount of CMC-Na with 99 mL of 40% glycerol aqueous solution at 65 °C. The two solutions were mixed evenly, frozen at −20 °C using circulating water multi-purpose vacuum pump (SHB-Ⅲ, Great Wall Scientific Industrial and Trade Co., Ltd., Zhengzhou, China), and subjected to three freeze–thaw cycles to obtain AEPE-PVA/CMC-Na hydrogels (AEPE composite hydrogel). The effects of all ratios of PVA solution (11%, 12%, 13%) and CMC-Na solution (0%, 0.5%, 1%, 1.5%) with on the drying resistance, swelling, water retention rate, moisture absorption rate, and gel fraction of the AEPE composite hydrogel were investigated using a single factor method.

### 2.9. Drying Resistance Analysis

A specified mass of AEPE composite hydrogel samples was placed in a Petri dish, exposed to air at room temperature for a set period, and weighed at 24 h and 72 h. The mass at each time point was recorded and the residual mass ratio was calculated.

### 2.10. Swelling Analysis

A specified mass of AEPE composite hydrogel samples was weighed and dried to a constant weight at 60 °C (m). The samples were then immersed in deionized water at 37 °C. At 72 h, the samples were removed, blotted to remove surface liquid with filter paper, and weighed (m′). The swelling ratio (SR) was calculated using the following formula:

(2) $$SR=\frac{m^{’}-m}{m}\times 100\%$$

### 2.11. Water Retention Rate Analysis

The dry AEPE composite hydrogel was weighed (m_1_), fully swollen in the PBS buffer, the surface water was removed, and it was weighed again (m_t_). The hydrogel was placed in a 37 °C incubator and weighed at regular intervals (m_2_). The 24 h water retention rate was calculated using the following formula:

(3) $$Water\;retention\;rate\left(\%\right)=\frac{m_{2}-m_{1}}{m_{t}-m_{1}}\times 100\%$$

### 2.12. Moisture Absorption Rate Analysis

A 35% gelatin gel was prepared by stirring gelatin in a 60 °C water bath, pouring it into a Petri dish, allowing it to solidify and cool, and weighing it (m_2_). The AEPE composite hydrogel was cut into the same shape and weight and attached to the surface of the gelatin gel. After standing at room temperature for 24 h, the gelatin gel was weighed again (m_3_). The moisture absorption rate of the hydrogel was calculated using the following formula:

(4) $$Moisture\;absorption\;rate(\%)=\frac{m_{3}-m_{2}}{m_{2}}\times 100\%$$

### 2.13. Gel Fraction Analysis

The wet AEPE composite hydrogel was dried in an oven to a constant weight (m), swollen to equilibrium in deionized water, and then dried at 37 °C to a constant weight (m’). The gel fraction (G) was calculated using the following formula:

(5) $$G=\frac{m^{’}}{m}\times 100\%$$

### 2.14. Characterization of AEPE Composite Hydrogel

The surface morphology of PVA/CMC-Na hydrogel and AEPE composite hydrogel was examined using a scanning electron microscope (MIRA, TESCAN, Shanghai, China). The rheological properties of AEPE composite hydrogel, including oscillation frequency and shear viscosity at 37 °C, were assessed using a rheometer (DHR-1, Waters, Milford, MA, USA).

### 2.15. In Vitro Release Profile

Different concentrations (6 mg/mL, 8 mg/mL, and 10 mg/mL) of AEPE composite hydrogel were immersed in 50 mL PBS and subjected to shaking. At intervals of 0.5, 1, 2, 3, 6, 9, 12, and 24 h, 1 mL of the medium was collected for analysis, and an equal volume of fresh PBS was added to maintain sink conditions. The cumulative release of the drug was measured using the gallic acid standard curve.

### 2.16. Biocompatibility and Cell Migration Ability Evaluation

The biocompatibility of the hydrogel materials was assessed using 3T6 cell viability, as described in the literature [24]. Cells were cultured in high-glucose DMEM medium at 37 °C with 5% CO_2_. Initially, 3T6 cells (1 × 10^5^/mL, 1 mL) were seeded in 6-well plates for 24 h. Cells were then incubated with hydrogel at different concentrations (1, 5, 10, 20, 40 mg/mL) for 24 h, with wells without hydrogel serving as blank controls. After incubation, the culture medium was removed, and 100 μL of MTT (5 mg/mL) was added to each well, followed by incubation at 37 °C for 4 h. The supernatant was removed, 200 μL of dimethyl sulfoxide was added, and the absorbance (OD) at 490 nm was measured using a microplate reader (800 TS, Nanjing BIO-SOAR Technology Co., Ltd., Nanjing, China). OD_T_ is the absorbance of the experimental group, OD_C_ is the absorbance of the control group, and OD_0_ is the absorbance of the blank group. The cell survival rate was calculated as follows:

(6) $$Cell\;survival\;rate\left(\%\right)=\frac{{OD}_{T}-{OD}_{0}}{{OD}_{C}-{OD}_{0}}\times 100\%$$

A scratch assay was used to evaluate the cell migration-promoting ability of AEPE composite hydrogel. The 3T6 cells were first seeded into 6-well plates, and a 200 μL pipette tip was used to create a scratch along the diameter of the well. Blank and drug-loaded hydrogels were dissolved in serum-free medium and added to the respective wells, with wells without hydrogel serving as blank controls. Detached cells were gently washed away with serum-free medium, and cell migration images were captured under an inverted fluorescence microscope (IX73, Olympus Corporation, Tokyo, Japan) at 12 h, 24 h, and 48 h. The cell migration rate was subsequently calculated.

### 2.17. Wound Healing Study

Back wounds in mice model were achieved following the method described by Sun with slight modifications [25]. Twelve mice were divided into four groups, with three mice per group: the control group, free AEPE group, blank hydrogel group, and AEPE composite hydrogel group. After anesthesia, hair was removed, and a 1 cm × 1 cm full-thickness wound was created on the back of each mouse. From day 0 to day 14, the control group received no treatment, while the free AEPE group was treated with a 10 mg/mL AEPE aqueous solution. The PVA/CMC-Na hydrogel sheets and AEPE composite hydrogel sheets were applied to the wounds in the blank hydrogel group and AEPE hydrogel group, respectively, every two days. The remaining wound area of the mice was photographed. The initial wound area of the mice was recorded as S_0_, and the wound area on day t was recorded as S_t_. The remaining wound area of the mice was calculated as follows:

(7) $$Remaining\;wound\;area\left(\%\right)=\frac{S_{t}}{S_{0}}\times 100\%$$

After 7 days of treatment, blood samples were collected from each group, and the serum concentrations of TNF-α, IL-1β, and IL-6 were measured using ELISA kit. After 14 days of treatment, the wound tissue was excised, fixed in 4% paraformaldehyde solution for 24 h, and subjected to histopathological analysis and histological analysis using H&E and Masson staining.

### 2.18. Statistical Analysis

All experiments were repeated three times. The experimental results are expressed as mean ± SD. One-way or two-way ANOVA was used to determine differences between samples. *p* < 0.05 was considered statistically significant. GraphPad Prism 9.5 software was used for data analysis.

## 3. Results and Discussion

### 3.1. Polyphenol Content and Antioxidant Activity of Different Solvent Extracts

**Figure 1:**
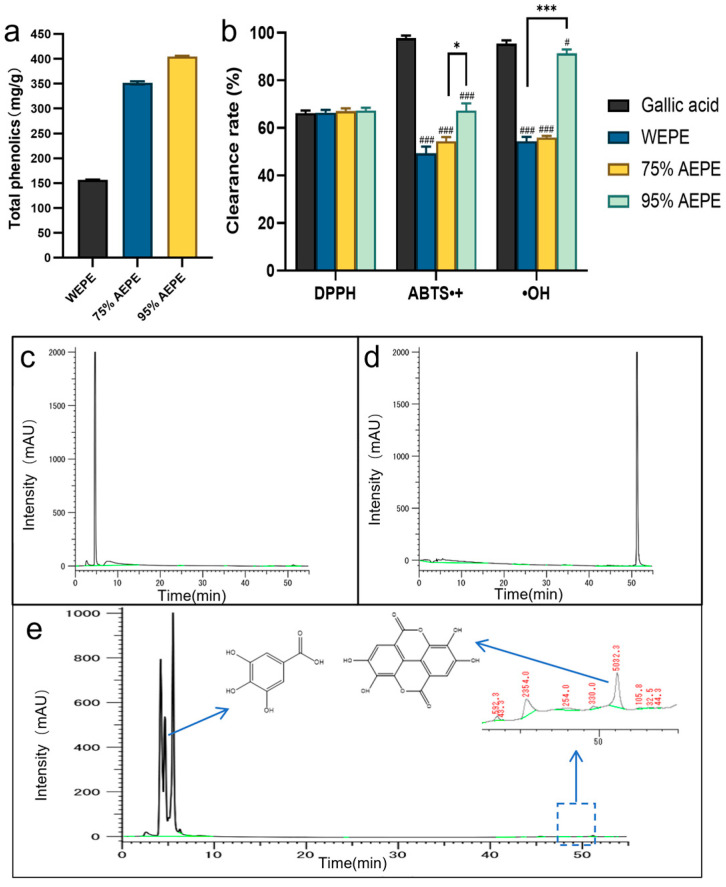
(**a**) Contents of total polyphenols in water extract (WEPE) and ethanol extract (AEPE) of Phyllanthus emblica; (**b**) scavenging rate of DPPH, ABTS^+^, and hydroxyl radical by Phyllanthus emblica officinalis extracts; HPLC of gallic acid (**c**), ellagic acid (**d**), and AEPE (**e**). # represents the difference between each solvent extraction and gallic acid (# *p* < 0.05, ### *p* < 0.001); * represents the difference between individual solvent extracts (mean ± SD, *n* = 3; * *p* < 0.05, *** *p* < 0.001).

The polyphenol content of WEPE and AEPE was measured, as shown in Figure 1a. The polyphenol content in AEPE extracted using 95% ethanol was 404.71 ± 1.77 mg/g, which was 2.58 times higher than that in WEPE. This suggests that the polyphenols in Phyllanthus emblica extract are more soluble in ethanol than in water.

The free radical scavenging rates of different solvent extracts of Phyllanthus emblica were compared using corresponding absorbance as an indicator [26,27,28], as shown in Figure 1b. The DPPH radical scavenging rates of different solvent extracts of Phyllanthus emblica were all above 60%, with no significant difference compared to gallic acid, indicating that both WEPE and AEPE have strong DPPH radical scavenging abilities. However, gallic acid exhibited a strong ABTS^+^ radical scavenging ability, reaching 97.71 ± 1.07%, while WEPE and AEPE had weaker scavenging abilities at the same concentration, with 95% AEPE being the highest at 67.31 ± 3.01%. Furthermore, the hydroxyl radical scavenging ability of 95% AEPE was close to that of gallic acid, reaching 91.39 ± 1.54%, which is much higher than that of WEPE and 75% AEPE at the same concentration, indicating that 95% AEPE has the strongest antioxidant capacity. Taken together, the results demonstrated that 95% AEPE has the strongest free radical scavenging ability, indicating its strong antioxidant capacity.

### 3.2. Active Ingredients in AEPE

The antioxidant activity of polyphenols can be classified into various types [29], which can be identified by HPLC analysis for the characterization of polyphenol species. The retention times (RTs) of gallic acid and ellagic acid were 4.7 and 51 min, respectively (Figure 1c,d). The ethanol extract of Phyllanthus emblica exhibited peaks at these RTs, indicating that the main polyphenols in AEPE were gallic acid and ellagic acid, and both of these phenolic compounds are often reported as having antioxidant effects on plants [30]. The content of gallic acid in Phyllanthus emblica found to be 16.46%, while the content of ellagic acid was only 0.012% (Figure 1e). In addition, gallic acid has the strongest free radical scavenging ability among polyphenols, indicating that the strong antioxidant effect is largely attributable to gallic acid [4]. Therefore, gallic acid was used as the detection index of antioxidant active components in Phyllanthus emblica in the subsequent characterizations.

### 3.3. Evaluation of Hydrogel Performance

**Figure 2:**
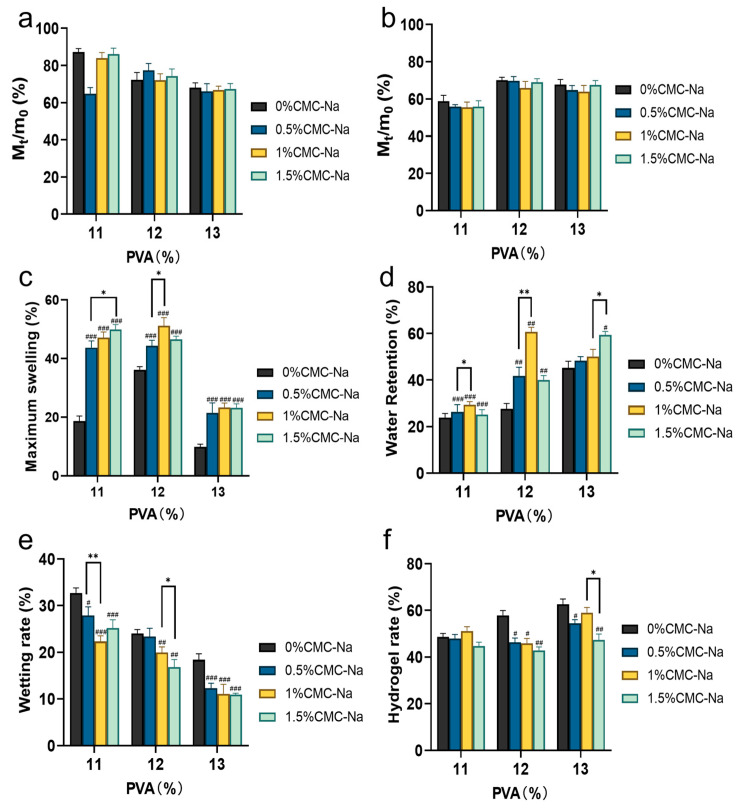
Evaluation of AEPE composite hydrogel performance: (**a**) residual weight rate of hydrogel with different ratios at 24 h and (**b**) 72 h; (**c**) maximum swelling rate of hydrogel with different ratios; (**d**) water retention of hydrogel with different ratios; (**e**) wetting rate of hydrogel with different ratios; (**f**) hydrogel fraction of hydrogel with different ratios. # represents the difference between CMC-Na with each content and 0% CMC-Na (# *p* < 0.05, ## *p* < 0.01, ### *p* < 0.001); * represents the difference between the CMC-Na of each content (mean ± SD, *n* = 3; * *p* < 0.05, ** *p* < 0.01).

The indexes of swelling rate, water retention rate, moisture delivery rate, and gel fraction of PVA/CMC-Na hydrogel were systematically evaluated [31,32]. The residual weights of AEPE composite hydrogel at 24 h and 72 h are presented in Figure 2a,b. Across the different hydrogel groups, weight differences were not statistically significant, with residual weights after 72 h still remaining at 55.47~70.07% of the initial values. This result indicates that the AEPE composite hydrogel exhibited considerable structural integrity and minimal water loss under various conditions. The maximum swelling rates of hydrogel made from different ratios of PVA and CMC-Na are illustrated in Figure 2c. Compared to pure PVA hydrogel, the composite hydrogel demonstrated a significantly enhanced swelling capacity. Notably, the hydrogel composed of 12% PVA and 1% CMC-Na showed the highest swelling rate of 51.21 ± 2.75%, indicating its superior ability to effectively absorb wound exudate.

The 24 h water retention rates of AEPE composite hydrogel with varying PVA and CMC-Na ratios were shown in Figure 2d. The pure PVA hydrogel exhibited a water retention rate of 45.19 ± 2.88%. However, the addition of 1% CMC-Na to 12% PVA significantly increased the water retention rate to 60.72 ± 1.98%, thereby enhancing the hydrogel’s ability to maintain a moist wound environment, which is critical for effective wound healing. The moisture absorption rates are depicted in Figure 2e, showing that increasing PVA content led to a decrease in moisture absorption due to tighter internal crosslinking. Specifically, the moisture absorption rate of 13% PVA hydrogel was reduced to 18.37 ± 1.28%. In comparison to single-component PVA hydrogel, composite hydrogels with different ratios exhibited significant variations in moisture absorption, indicating that higher concentrations of PVA and CMC-Na in the hydrogel matrix reduce moisture absorption capacity.

The gel fractions of AEPE composite hydrogel with different ratios are illustrated in Figure 2f. At low PVA concentrations, the gel fractions showed no significant differences across hydrogel groups. However, increasing the PVA content to 13% resulted in the formation of a more stable and tightly crosslinked structure, achieving a gel fraction of 62.62 ± 2.29%. Conversely, increasing the CMC-Na concentration disrupted the hydrogel’s internal structure, thereby reducing crosslinking density and ultimately decreasing the gel fraction [33]. Overall, the incorporation of 1% CMC-Na was found to be optimal, striking a balance between maintaining structural stability and favorable physical properties.

### 3.4. Quality Evaluation

**Figure 3:**
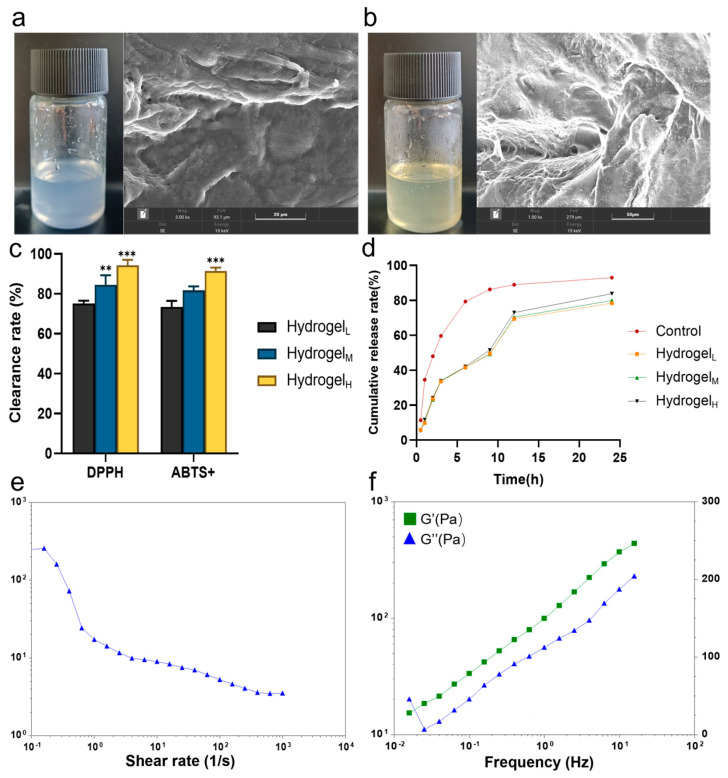
Scanning electron microscopy images of (**a**) PVA/CMC-Na hydrogel and (**b**) AEPE composite hydrogel (** *p* < 0.01, *** *p* < 0.001); (**c**) free radical scavenging of the AEPE composite hydrogel; (**d**) in vitro release curves of PVA/CMC-Na hydrogel with different gallic acid contents over 24 h. Rheological tests: (**e**) shear viscosity and (**f**) frequency scanning.

The appearances of PVA/CMC-Na hydrogel and AEPE composite hydrogel are depicted in Figure 3a,b. The addition of CMC-Na had minimal impact on the transparency, color, and overall appearances of the hydrogel. Under scanning electron microscopy (SEM), the drug-loaded composite hydrogel exhibited a dense structure, consistent with the morphology observed in the blank hydrogel.

The antioxidant capacity of AEPE composite hydrogel, evaluated through DPPH and ABTS^+^ radical scavenging assays, is shown in Figure 3c. The high-concentration hydrogel group had a gallic acid content of 8.23 mg, achieving a DPPH radical scavenging rate of 94.31 ± 1.17% and an ABTS^+^ radical scavenging rate of 91.47 ± 1.64%. These results highlight the robust antioxidant capacity of the AEPE composite hydrogel, underscoring its strong potential for scavenging free radicals [34]. Figure 3d presents the release profiles of three AEPE composite hydrogel with varying gallic acid contents were similar. The cumulative release rates at 12 h for the low-, mid-, and high-content gallic acid groups (4.94 mg, 6.58 mg, and 8.23 mg, respectively) were 68.12 ± 0.96%, 69.36 ± 0.67%, and 73.11 ± 2.15%. Compared to the control group, AEPE composite hydrogel exhibited a slower release of AEPE in PBS buffer (pH = 7.4), regardless of the gallic acid content.

The shear viscosity test results, shown in Figure 3e, indicate that the AEPE composite hydrogel possesses excellent shear-thinning characteristics, which allows for smooth application to wounds under stress and firm attachment when not under stress. Additionally, the frequency scanning results (Figure 3f) show that the storage modulus G’ of AEPE composite hydrogel is greater than the corresponding loss modulus G’’ across the frequency range of 0.01–100 Hz, indicating that elasticity exceeded viscosity. This suggests that the hydrogel exhibits the properties of a viscoelastic solid gel, providing a stable structure suitable for wound healing applications.

### 3.5. Biological Safety Evaluation

**Figure 4:**
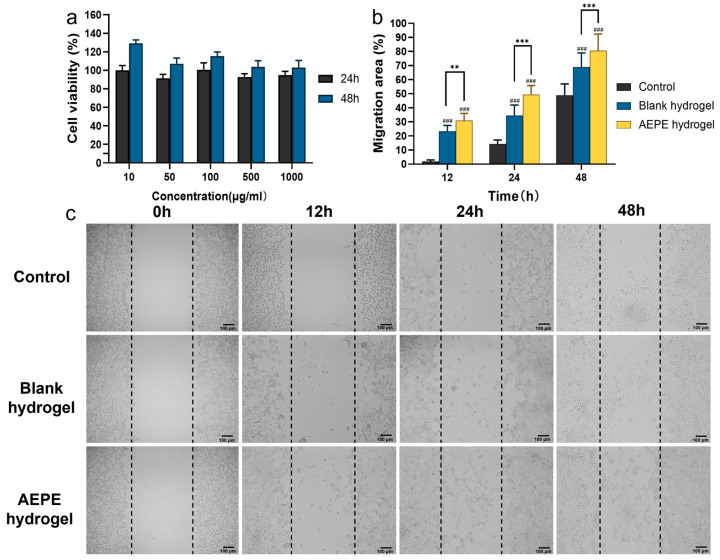
(**a**) Cell viabilities of 3T6-Swiss albino mice treated with free film for 24 h and 48 h; (**b**) rates and (**c**) fibroblast migration behaviors after blank hydrogel with AEPE hydrogel. # represents the difference between hydrogel of each group and control group (### *p* < 0.001); * represents the difference between the hydrogels of each group (mean ± SD, *n* = 3; ** *p* < 0.01, *** *p* < 0.001).

The cell compatibility and cytotoxicity results of hydrogel in 3T6 cells at different time points were shown in Figure 4a. Within the investigated concentration range, cell survival rates slightly decreased but remained above 90%, indicating that cells could normally proliferate and grow in the presence of blank hydrogel. These findings confirmed the excellent biocompatibility of blank hydrogel. Furthermore, as the incubation time increased, the cell survival rate exceeded 100% at the same concentration, suggesting that blank hydrogel might possess growth-promoting properties. Figure 4b,c compare the cell migration of the blank hydrogel and AEPE hydrogel. The cell migration rates in both blank hydrogel and AEPE hydrogel were significantly higher than those in the control group (*p* < 0.001). Furthermore, the AEPE composite hydrogel demonstrated the strongest potential in promoting cell migration, showing significantly enhanced effects compared to the blank hydrogel. This result indicated that the AEPE composite hydrogel may possess the potential to accelerate wound healing.

### 3.6. Wound Healing Evaluation

**Figure 5:**
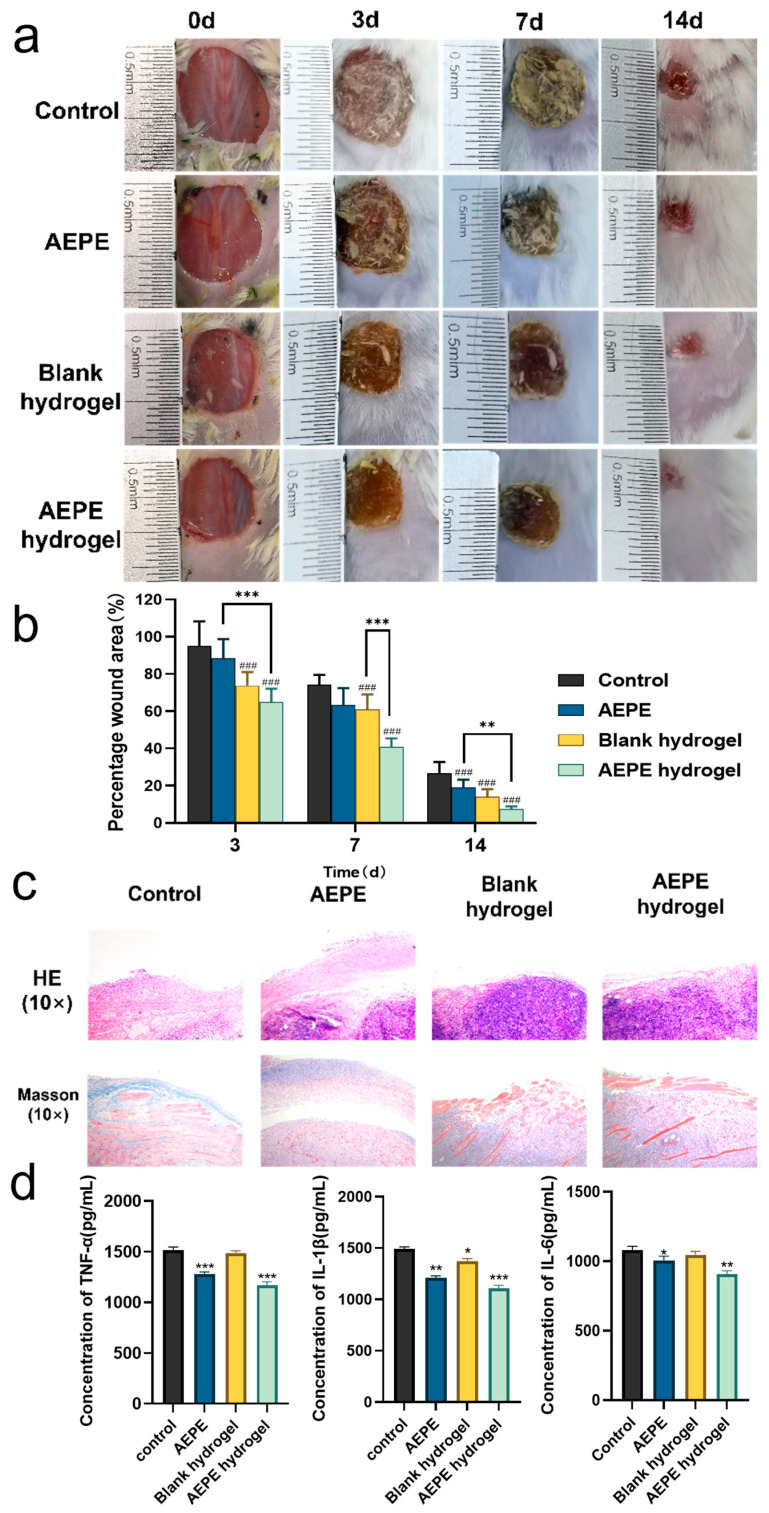
(**a**) The wound healing processes recorded for the rats by using different treatments (control, AEPE, blank hydrogel, and AEPE hydrogel) at various times (0–14 days); (**b**) time-dependent wound healing area after different treatments. (**c**) Histological analysis of tumor sections on day 14 after different treatments. (**d**) TNF-α, IL-1β, and IL-6 concentrations, expressed as pg/mL, in the control and treatment groups (d7). # represents the difference between the treatment groups and the control group (### *p* < 0.001); * represents the difference between treatment groups (mean ± SD, *n* = 3; * *p* < 0.05, ** *p* < 0.01, *** *p* < 0.001).

The wound healing progression in mice treated with different groups is shown in Figure 5a. On day 0, the initial wound sizes across all four groups were approximately the same, confirming the consistency of the experimental setup. During the treatment period, the wound areas in all four groups gradually decreased. However, due to the presence of more wood chips in the wounds of the control and free AEPE groups compared to the gel dressing groups, wound healing was relatively inhibited. At all observed time intervals (3–14 days), the wound areas in both the blank hydrogel group and the AEPE hydrogel group were significantly smaller than those in the control group. On day 7, the wound area in the AEPE hydrogel group was notably smaller than that in the blank hydrogel group, indicating a faster wound recovery rate due to the antioxidant activity of Phyllanthus emblica (Figure 5b). These results demonstrated that the hydrogel protected wounds from external stimuli, with the AEPE hydrogel being more efficacious in promoting wound healing than the blank hydrogel.

To further characterize the wound healing in each treatment group, morphological studies were conducted on newly formed wounds to observe inflammatory cell infiltration, blood vessel and hair follicle formation, collagen deposition, and new epithelial tissue [35]. As shown in Figure 5c, HE staining revealed inflammatory cell infiltration in all groups, with significantly fewer inflammatory cells in the free AEPE group, blank hydrogel group, and AEPE hydrogel group. These groups also showed the presence of epithelial layers. Specifically, the two gel-treated groups had intact epithelial layers and fully closed wounds, whereas the epidermis in the free AEPE group was not fully closed, indicating that it was still in the re-epithelialization stage. Hair follicles and other skin appendages, as well as blood vessels, were observed in the AEPE hydrogel group compared to the blank hydrogel group, with a clear epithelial layer interface and denser tissue recovery. This result indicated that the AEPE hydrogel could gradually release AEPE, promoting wound healing and inhibiting scar formation. Masson staining reflected the amount of collagen and granulation tissue formation in different treatment groups and collagen was found to be beneficial for wound healing [36]. Compared with the blank group, the collagen in each treatment group increased, with improved density evident in granulation tissue growth, and epithelial implantation visible at the edge. The AEPE hydrogel had clearer and denser granulation tissue with extensive blood vessel formation. This result demonstrated that the AEPE hydrogel could release AEPE to reduce inflammation, enhance the wound microenvironment, promote epithelial cell proliferation, and protect the wound to promote angiogenesis, thereby accelerating tissue remodeling. Delayed wound healing and the chronic nature of the wound are directly related to ongoing inflammation. We analyzed the concentrations of TNF-α, IL-1β, and IL-6 as markers of inflammatory response [37]. Figure 5d shows that the levels of TNF-α and IL-1β in the AEPE group and the AEPE hydrogel group were significantly lower than those in the control group, while the level of IL-6 in the AEPE hydrogel group was significantly lower than that in the control group.

## 4. Conclusions

In summary, our study has unveiled the remarkable antioxidant properties of Phyllanthus emblica extract, which can be attributed to its rich polyphenolic content, particularly gallic acid. The composite hydrogel of Phyllanthus emblica extract (AEPE composite hydrogel) has further enhanced its effectiveness for wound healing. The optimized formulation of the AEPE composite hydrogel, tailored with specific contents of PVA and CMC-Na, exhibits a porous structure, along with exceptional swelling and water retention capabilities, which are pivotal for prolonged drug release and enhanced wound healing. Importantly, the AEPE composite hydrogel has been shown to significantly accelerate cell migration and proliferation. Additionally, in vivo models have demonstrated the AEPE composite hydrogel significantly enhances collagen formation and angiogenesis, mitigates inflammation, and accelerates tissue regeneration, thereby underscoring its benefits in wound healing. Notably, our research furthers the therapeutic potential of Phyllanthus emblica as a natural antioxidant in wound healing applications, leveraging its robust bioactivity and superior biocompatibility. Furthermore, the straightforward and scalable method of preparing AEPE composite hydrogels is highly advantageous for industrial-scale production. This not only facilitates the broader clinical and commercial adoption of AEPE composite hydrogel but also offers a natural, potent, and viable alternative for wound therapy.

## Data Availability

The original contributions presented in this study are included in the article, and further inquiries can be directed to the corresponding authors.
